# Invited Commentary: Neuraxial Anaesthesia for Appendectomy: Another Tool in the Toolbox

**DOI:** 10.1007/s00268-021-06002-w

**Published:** 2021-08-21

**Authors:** Folke Hammarqvist, Mark Schumacher

**Affiliations:** Department of Emergency Surgery and Trauma, Department of Clinical Science, Intervention and Technology (CLINTEC), Unit of Surgery, Karolinska University Hospital Stockholm, Karolinska Institute, Stockholm, Sweden

In this number of World Journal of Surgery, the use of Neuraxial Anaesthesia (NA, spinal, or epidural anaesthesia) for appendectomy is discussed and compared to the use of general anaesthesia (GA) [1]. The study material originates from an earlier multicentre study “Opioids After Surgery in the United States Versus the Rest of the World: The International Patterns of Opioid Prescribing (iPOP) Multicenter Study” published in 2020 in Annals of Surgery [2]. Participants were drawn from around the globe with a large proportion coming from Brazil, Colombia, China, and Thailand, where almost half of the patients in the present study underwent appendectomy with NA. Although there were potential differences in material, age, and perioperative procedures, a multivariable regression analysis adjusted for age, gender, BMI, smoking, emergency status, and country showed fewer complications in the NA group. These were categorized as wound-related, infectious, non-infectious or other complications. Moreover, the authors report a reduced length of stay and lower pain severity in the NA group.

The favourable effects of NA may be explained by fewer side effects, a lesser impact on pulmonary function, reduced nausea and vomiting, and a reduction in postoperative pain. NA has proved its part in the multimodal Enhanced Recovery After Surgery programs (ERAS) which promote early mobilization and oral intake, and in which NA and local anaesthesia are important cornerstones [3].

Appendectomy is traditionally performed under general anaesthesia in many countries but there are obvious variations in circumstances and resources around the globe where NA may prove to be a useful alternative. The feasibility of performing laparoscopic appendectomy in NA has been communicated earlier [4].

Despite its scientific underpinnings there continues to exist a fair amount of dogma in everyday surgical practice: We do it this way because that is the way we have always done it. Progress in surgical techniques and practices often comes about when the surgeon is backed into a corner by lack of time or resources, such as during war and natural disasters. Careful observation and analysis of such situations can yield valuable insights that contribute to major improvements in healthcare.

The fact that NA is used more commonly in some countries may be explained by socio-economic factors since NA is associated with lower operating room costs as compared to GA. NA may also reduce the need for resources related to ventilation and perioperative monitoring.

This study gives rise to some thoughts that are summarized below.

The Covid-19 pandemic currently affects almost every aspect of healthcare. With this in mind, the risk to the operating room team from the contaminated aerosols produced by intubation, positive pressure ventilation, and laparoscopy may be reduced by performing suitable open operations with NA instead of GA. Current guidelines still recommend the use of laparoscopy as long as the procedure can be safely performed, however, since laparoscopy contributes to a reduced length of stay and thereby reduces the overall strain on hospital resources.

It may be possible to show additional benefits of NA for appendectomy if particular patient groups are identified and studied. NA could potentially prove to be superior therapy for patients who are at risk for complications of intubation, such as those with severe pulmonary disease or difficult airway.

The likelihood for appendicitis should be high when NA is chosen since intraoperative options will be quite limited with regard to further exploration of the abdominal cavity or to performing a different or more extensive procedure than was originally planned. Accurate diagnosis with preoperative ultrasound or CT along with careful clinical examination and laboratory tests will be essential to ensuring the best results from NA.

Unique situations will inevitably arise from time to time in the practice of surgery. One is reminded of the case of Leonid Rogozov, a surgeon stationed in Antarctica who was obligated to remove his own appendix. No doubt he would have been grateful for NA had it been available to him [5].

To compare NA and GA in a prospective study would be of great interest and could significantly contribute to the advancement of perioperative care and emergency surgery.
